# Phone-based monitoring to evaluate health policy and program implementation in Kenya

**DOI:** 10.1093/heapol/czab029

**Published:** 2021-03-16

**Authors:** Paul G Ashigbie, Peter C Rockers, Richard O Laing, Howard J Cabral, Monica A Onyango, John Mboya, Daniella Arends, Veronika J Wirtz

**Affiliations:** Department of Global Health, Boston University School of Public Health, 801 Massachusetts Avenue, Boston, MA 02118, USA; Department of Global Health, Boston University School of Public Health, 801 Massachusetts Avenue, Boston, MA 02118, USA; Department of Global Health, Boston University School of Public Health, 801 Massachusetts Avenue, Boston, MA 02118, USA; Faculty of Community Health Sciences, School of Public Health, University of the Western Cape, Robert Sobukwe Road, Bellville 7535, Cape Town, Republic of South Africa; Department of Biostatistics, Boston University School of Public Health, 801 Massachusetts Avenue, Boston, MA 02118, USA; Department of Global Health, Boston University School of Public Health, 801 Massachusetts Avenue, Boston, MA 02118, USA; Innovations for Poverty Action, Sandalwood Lane, Westlands, Nairobi, Kenya; Faculty of Sciences, Department of Pharmaceutical Sciences and School of Pharmacy, Utrecht University, Universiteitsweg 99, 3584 CG Utrecht, The Netherlands; Department of Global Health, Boston University School of Public Health, 801 Massachusetts Avenue, Boston, MA 02118, USA

**Keywords:** Telephone interviews, in-person interviews, price, availability, health facility, household

## Abstract

Monitoring and evaluating policies and programs in low- and middle-income countries are often difficult because of the lack of routine data. High mobile phone ownership in these countries presents an opportunity for efficient data collection through telephone interviews. This study examined the feasibility of collecting data on medicines through telephone interviews in Kenya. Data on the availability and prices of medicines at 137 health facilities and 639 patients were collected in September 2016 via in-person interviews. Between December 2016 and December 2017, monthly telephone interviews were conducted with health facilities and patients. An unannounced in-person interview was conducted with respondents to validate the telephone interview within 24 h. A bottom-up itemization costing approach was used to estimate the costs of telephone and in-person data collection. In-depth interviews were conducted with data collectors and respondents to explore their perceptions on both modes of data collection. The level of agreement between data on medicines availability collected through phone and in-person interviews was strong at the health facility level [kappa = 0.90; confidence interval (CI) 0.88–0.92] and moderate at the household level (kappa = 0.50, CI 0.39–0.60). Price data from telephone and in-person interviews showed strong intra-class correlation at health facilities [intra-class correlation coefficient (ICC) = 0.96] and moderate intra-class correlation at households (ICC = 0.47). The cost per phone interview at health facilities and households were $19.73 and $16.86, respectively, compared to $186.20 for a baseline in-person interview. Participants considered telephone interviews to be more convenient. In countries with high cell phone penetration, telephone data collection should be considered in monitoring and evaluating public health programs especially at health facilities. Additional strategies may be needed to optimize this mode of data collection at the household level. Variations in cell phone ownership, telecommunication network and data collection costs across different settings may limit the generalizability of the findings from this study.

**KEY MESSAGES**

- Telephone interviews yielded valid data on medicines at health facility and household levels
- Telephone interviews cost less, compared to in-person interviews and can yield high response rates
- Most of the facilitators and challenges for phone and in-person data collection are similar
- In settings with high cell phone penetration rate, phone interviews can be used to collect low cost validated data to monitor and evaluate policies and programs

## Introduction

Evaluating the effect of policies and programs in low- and middle-income countries (LMICs) can be challenging due to the lack of routine data. High mobile phone ownership in LMICs presents an opportunity for efficient data collection through phone interviews both for one-off surveys and continuous surveillance. In high-income countries, this mode of data collection has been used for a diverse set of purposes, including monitoring illicit drug use and consumer market research (Gfroerer and Hughes, 1991; Greenfield, Midanik and Rogers, 2000; Turner *et al.*, 2005; Szolnoki and Hoffmann, 2013). However, there is limited evidence on the use of phone interviews for data collection in LMICs to evaluate policy and programmatic interventions. This study aims to examine the feasibility of collecting data on medicine availability and price through phone surveys in semi-urban and rural communities Kenya. The objectives of this study were:

- to validate a method for the collection of information on health facility and patient medicines through phone interviews;
- to compare the costs associated with collecting data on medicines through phone interviews and in-person interviews; and
- to describe the perceptions of data collectors and respondents on each mode of data collection.

In-person interviews have been the primary method of data collection in global health research, particularly in LMICs (Croke *et al.*, 2012; Ballivian *et al.*, 2015). Phone interviewing holds great potential for global health research because it is less expensive, less time consuming and offers flexibility for larger sample size studies (Greenleaf *et al.*, 2017). The dramatic increase in mobile phone ownership in LMICs in recent years has made mobile phone surveys much more attractive in these countries (Greenleaf *et al.*, 2017; Hyder *et al.*, 2017). In 2018, there were 100 mobile phone subscriptions per 100 people in LMICs, which is close to the worldwide subscription rate of 104 per 100 people (Gibson *et al.*, 2017; World Development Indicators, 2018). In the same year, the subscription of fixed phones stood at 13 per 100 worldwide and 8 in LMICs (World Development Indicators, 2018).

Despite the high mobile phone ownership in LMICs there has been limited use of phones for surveys and continuous surveillance in these settings. A systematic review conducted by Gibson *et al.* on the use of mobile phone surveys (MPS) for collecting population-level data in LMICs found only 19 surveys, 8 each in Latin America and sub-Saharan Africa, 2 in Asia and 1 in the Middle East (Gibson *et al.*, 2017). These countries, which need quality data at low cost, often have less safe environments and limited infrastructure (road network) needed for in-person interviews (Demombynes *et al.*, 2013; Etang-Ndip *et al.*, 2015; Greenleaf *et al.*, 2017).

A variety of technologies may be used to collect data through phone interviews (Croke *et al.*, 2012; Ballivian *et al.*, 2015; Gibson *et al.*, 2017). Two of these technologies have been used more frequently in LMICs—short messaging services (SMS) and voice phone calls (Dillon, 2010; Gibson *et al.*, 2017). Compared to phone calls, SMS may be of lower cost but have several limitations including the skills involved in typing and reading text messages, the amount of data that can be collected, among others (Dillon, 2010). Phone calls require less skill and allow for gathering more data. Human operator or computer-assisted phone interview (CATI) in which respondents are interviewed through a phone call and the interviewers utilize a software program to record the survey responses, allows for efficient and fast processing for data cleaning and analysis (Gibson *et al.*, 2017). The choice of phone technology depends on the type of study, including the nature of the questions, the amount of data to be provided, respondents and cost (Croke *et al.*, 2012; Ballivian *et al.*, 2015). Phone calls may be the best option for studies requiring long responses to many questions; and studies involving older and less educated respondents who may have difficulty working with text-based phone responses.

Though phone data collection has the potential to reduce social desirability bias that is common in in-person interviews, Ballivian *et al.* (2015) identified three main challenges associated with phone data collection compared to in-person interviews: obtaining samples that are representative of the study population, ensuring adequate response rates and collecting good quality data. Selection bias may be associated with phone data collection because people who are less familiar with the phones will have more difficulty using it to provide data (Ali *et al.*, 2017). Unreliable network coverage not only affects response rates but may also introduce bias in the study (Dillon, 2010; Demombynes *et al.*, 2013; Etang-Ndip *et al.*, 2015; Himelein and Kastelic, 2015).

Several studies have compared the validity of data collected from phone interviews, though these studies were not on price and availability of medicines (Roccaforte *et al.*, 1992; Aziz and Kenford, 2004; Janssen *et al.*, 2010; Paing *et al.*, 2010; Hajebi *et al.*, 2012; Szolnoki and Hoffmann, 2013; Silva *et al.*, 2014). Two studies involving the use of SMS technology to collect routine data on stock levels of the anti-malarial drug, artemether-lumefantrine and rapid diagnostic tests at health facilities reported data accuracy of 79–94% (Barrington *et al.*, 2010; Githinji *et al.*, 2013). Furthermore, there is consistent evidence suggesting phone interviews are cheaper compared to in-person interviews (Ballivian *et al.*, 2015). Various studies reported the CATI ranging from $4.10 to $7.30 compared to $35.96 to $150 for in-person interviews (Dillon, 2010; Croke *et al.*, 2012; Ballivian *et al.*, 2015; Mahfoud *et al.*, 2015). The moderate-to-high validity and low cost of phone data illustrate the high potential for this mode of data collection.

According to a nationally representative survey of 2011, each household in Kenya owns about 2.4 mobile phones, with 80% of Kenyans having their own mobile phones (Mitullah *et al.*, 2011). Though 7% of Kenyans reported never using a mobile phone, 81% said they make at least one call a day using their mobile phones. Phone ownership rates above 80% are acceptable for reliable surveys to be conducted over the phone (Croke *et al.*, 2012).

To our knowledge, this study is the first mobile phone serial survey on medicines availability and price not only in Kenya but in LMICs. If high response rates can be achieved and accurate price and availability data can be collected by phone, incorporating this into the evaluation of access to medicines programs in LMICs will reduce the cost of data collection while maintaining the same data validity as in-person data collection. This will make monitoring and evaluating access to medicine programs more feasible.

## Methods

### Study sites

This study was part of an evaluation of the impact of a low-cost medicines access program (instituted by Novartis/Sandoz Pharmaceuticals and called *Novartis Access*) on the availability and price of medicines for non-communicable diseases (NCDs) at health facilities and households in the Kenya (Rockers *et al.*, 2019). This evaluation (registered with ClinicalTrials.gov—NCT02773095), was a cluster randomized controlled trial that took place in eight counties—Embu, Kakamega, Kwale, Makueni, Narok, Nyeri, Samburu and West Pokot which have been randomized into four control and four intervention counties. The selection of these counties had been described by Rockers *et al.* (2016).

### Data collection

Evaluation of *Novartis Access* baseline data on availability and prices of medicines were collected through in-person surveys at health facilities (public, private not-for-profit and private for-profit) and a random sample of households with NCDs in October 2016 (Rockers *et al.*, 2016). During enrolment at baseline, phone numbers were collected and participants consented to the possibility of being called by phone to collect the same data collected in-person.

### Telephone interviews

Routine telephone surveillance on the availability and prices of medicines took place from December 2016 to December 2017. Data collectors were trained on key concepts on price and availability of medicines, ensuring data quality, ethics of data collection, phone etiquette, maintaining the confidentiality of respondents, administering informed consent and collecting data using CATI. Data were collected using the survey instruments programmed on a tablet, with the software application Survey CTO, version 2.50 (SurveyCTO [Technology for digital data collection], 2017). The study instruments were pilot tested by the trained data collectors and revised based on the feedback received from the pilot test (Evaluation of Novartis Access, no date). Telephone interview data collection from households and health facilities are described below.

#### Household data collection

A random sample of 400 of the households that took part in baseline data collection (representing 62.6% of 639 household participants) was included in the telephone surveillance. This sample size was to maximize the surveillance sample while also keeping enough households out of the surveillance to allow us to investigate potential changes in behaviour induced by the phone calls. We had 80% power to detect a 10-percentage point increase in the probability of having medicines at home due to the phone calls. Replacement samples were drawn for household respondents who dropped out of the surveillance. A rotating one-third sample of the 400 households was surveyed by phone each month. Data were collected via phone, on the availability of the NCD (asthma, breast cancer, diabetes and cardiovascular disease) medicines prescribed for these patients, the price at which these medicines were purchased, and where they were purchased (public hospital, public health centre/clinic, non-profit hospital/clinic, private for-profit hospital and private chemist). Respondents were given 50 KES (about $0.50) of airtime for each interview (Evaluation of Novartis Access, no date).

#### Health facility data collection

Data were collected through telephone interviews with all of the health facilities taking part in the *Novartis Access* evaluation. Data were collected monthly on 25 medicines (all medicines for NCDs with the exception of amoxicillin dispersible tablets). An additional list of 22 medicines was divided into three groups (Supplementary Appendix S1), and data were also collected on the medicines in each of these groups every 3 months. This was done to minimize the burden of data collection on health facilities. Health facilities were given 100 KES (about $1.00) in airtime per interview.

#### In-person validation interviews

For both health facility and household phone interviews, an unannounced in-person interview was conducted with a 10% subsample of respondents to validate the phone interview within 24 h of the phone-based interview. For ease of follow-up, this 10% sub-sample was randomly selected from two *Novartis Access* control (Embu and Kakamega) and two intervention (Makueni and Nyeri) counties.

### Costs of phone and in-person interviews

A bottom-up itemization costing approach was used to assess costs based on standard economic methods (Edejer *et al.*, 2003). Costs were estimated from the perspective of researchers (data collection). Information on all the major cost components, including phone airtime, cost of transportation and personnel costs etc. were collected by reviewing study records, standard operating procedures, financial reports and budgets. Costing was done separately for health facility phone interviews, household phone interviews, in-person validation interviews, baseline in-person interviews and listing of health facilities and households (which preceded baseline data collection). Baseline data collection costs could not be disaggregated into health facility and household costs since the same resources and personnel were dedicated to both. For the same reasons, listing costs and costs associated with in-person validation interviews could not be separated into household and health facility costs. Onetime upfront equipment costs for telephone surveillance were divided by 36 months (assuming a hypothetical surveillance duration of 3 years) to get the cost per month. Administrative costs were estimated as 19% of all direct costs, and overhead costs were 15% of all direct costs (including administrative costs). These estimates are based on the routine administrative and indirect costs incurred by the Kenya office of the organization that managed data collection.

### Qualitative data collection

The aim of the qualitative data collection was to understand the perceptions of both data collectors and study participants on data collection through telephone and in-person interviews. Qualitative data were collected between April and July 2017 by trained data collectors led by a researcher who had substantial experience in qualitative data collection and analysis. In-depth interviews (IDIs) were conducted with all data collectors, and a purposive sample of study participants from households and health facilities using semi-structured interview guides. Only household and health facility study participants who had participated in both phone surveillance data collection and in-person validation interviews were interviewed.

The interview guides covered the experience of respondents collecting or providing data via phone or in-person interviews, the facilitators and challenges associated with both modes of data collection, and their perceptions on the accuracy of data collected or provided.

After a follow-up, in-person data collection to audit data collected via a phone interview, the consent of the household and health facility study participants to take part in this qualitative data collection was sought. With the consent of the study participants, the interviews were audio recorded. Each interview lasted for about 30–45 min. The interviews with data collectors and facility-level study participants were conducted in English, while interviews with household participants were conducted in local languages.

## Data analysis

### Validity of data from phone interviews

Data were analysed using SAS version 9.4 (The SAS Institute Inc.) ([SAS/STAT] software, 2002). The intra-class correlation coefficient (ICC) was used to assess the validity of price, quantity and strength of medicine purchased data, collected through phone interviews. ICC measures the variance between pairs of observations, calculated as a proportion of the total variability across all observations (Ranganathan *et al.*, 2017). For each of these continuous variables, we also used the Bland–Altman plot to display the relationship between pairs of data collected from in-person and phone interviews (Ranganathan *et al.*, 2017). The level of agreement between medicine availability, availability of recommended pack size and place of purchase as assessed through phone interviews and in-person interviews were compared using the kappa statistic (McHugh, 2012). Since phone monitoring might affect the availability of medicines especially in households, baseline and midline *Novartis Access* evaluation data on the availability of medicines were compared between the households monitored by phone and those not monitored to determine the possible effect of the phone monitoring on household behaviour. Logistic regression was used to determine the effect of phone monitoring on the availability of medicines.

### Cost analysis

Microsoft Excel 2016 was used to compute cost estimates. A full cost analysis was conducted. The cost per interview was estimated for health facility phone interviews, household phone interviews, in-person validation interviews (aggregate for both facility and household) and baseline in-person data collection (aggregate for both facility and households).

### Qualitative data analysis

Qualitative data obtained was translated to English where necessary and transcribed. Data were analysed thematically using NVivo 11 QSR, assigning codes based on themes and categories of data observed.

## Results

### Background characteristics of study participants

A total of 421 household respondents and 138 health facilities participated in the phone surveillance. There were more female household participants than males (68.8 vs 31.2%) and <6% had at least a college-level education. In-person validation interviews were conducted with 105 households and 65 facilities. Tables 1 and 2 show the demographic characteristics of respondents.

**Table 1:** Background characteristics of household study participants

	Characteristics of household respondents			
	**All phone interviews** **(8 counties)** *N* = **410**	**Visited in-person (4 counties)** *N* = **105**	**Not visited in person (4 counties)** *N* = **158**	*P*-values (visited vs not visited)
Age in years				0.7900
Mean (range)	58.1 (18–101)	61.9 (30–94)	61.4 (19–101)	
Education level	*n* (%)	*n* (%)	*n* (%)	0.3307
Preschool (<1 year completed)/none	110 (26.8)	20 (19.1)	39 (24.7)	
Primary school (not completed)	105 (25.6)	27 (25.7)	50 (31.7)	
Primary school	88 (21.5)	32 (30.5)	29 (18.4)	
Secondary school	80 (19.5)	21 (20.0)	33 (20.9)	
Higher than secondary school	23 (5.6)	4 (3.8)	6 (3.8)	
Vocational School (Post primary)	4 (1.0)	1 (1.0)	1 (0.6)	
Gender	*n* (%)	*n* (%)	*n* (%)	0.8970
Male	128 (31.2)	28 (26.7)	117 (74.1)	
Female	282 (68.8)	77 (73.3)	41 (26.0)	
Wealth quintile	*n* (%)	*n* (%)	*n* (%)	0.5436
Quintile 1	76 (18.5)	9 (8.6)	19 (12.0)	
Quintile 2	94 (22.9)	23 (21.9)	36 (22.8)	
Quintile 3	88 (21.5)	24 (22.9)	45 (28.5)	
Quintile 4	67 (16.3)	20 (19.1)	25 (15.8)	
Quintile 5	85 (20.7)	29 (27.6)	33 (20.9)	

*N*, number of respondents (not number of interviews); NA, not applicable.

Validation visits took place in Embu, Kakamega, Makueni and Nyeri counties.

**Table 2:** Types of health facility participants

	Health facility respondents % (*n*)			
	All phone interviews	Visited in person	Not visited in person	*P*-values (visited vs not visited)
Level of care	*N* = 124 *n* (%)	*N* = 62 *n* (%)	*N* = 23 *n* (%)	0.5879
Level 2 (Dispensaries)	75 (60.5)	56.5 (35)	16 (69.6)	
Level 3 (Health centres)	20 (16.1)	11 (17.7)	3 (13.0)	
Level 4 (County referral hospitals)	24 (19.4)	13 (21.0)	4 (17.4)	
Level 5 (Teaching and referral hospitals)	5 (4.0)	3 (4.8)	0 (0.0)	
Provider type	*N* = 130 n (%)	*N* = 65 *n* (%)	*N* = 26 *n* (%)	0.1525
Public	56 (43.1)	28 (43.1)	7 (26.9)	
Private non-profit	74 (56.9)	37 (56.9)	19 (73.1)	

NA, not applicable.

The mean age of all household study participants was 58.1 years with a range of 18–101. The majority (60.5%) of health facilities were level 2 facilities (dispensaries). At least 15 health facilities participated from each county with the exception of Kwale, Samburu and West Pokot where 8, 6 and 10 facilities, respectively participated.

### Validity of data from phone interviews

**Figure 1:**
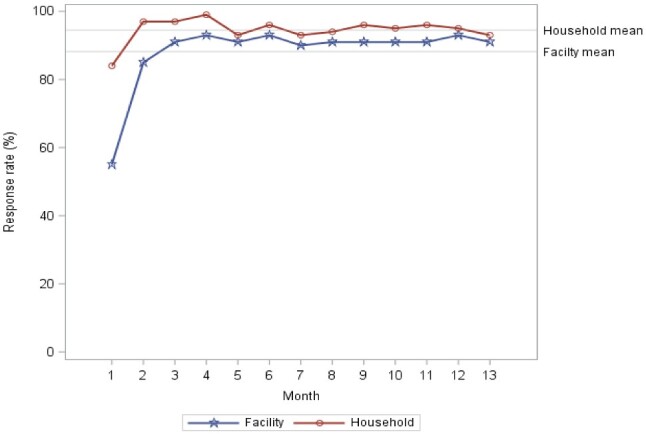
Response rates for phone interviews by month of data collection.

The mean response rate for phone interviews with health facilities was 88.2%. For households, the mean response rate was 94.5%. Figure 1 shows the response rates achieved over time. Phone interviews with facilities and households took 30.9 min and 12.8 min, respectively, compared to 14.9 min and 8.3 min for in-person interviews. For both modes of data collection at the facility and household levels, no clear trends were observed in changes in interview durations over time (Supplementary Appendices S2 and S3).

Supplementary Appendix S4 presents the response rates by county. Though response rates were generally high across all counties, Samburu county had the lowest response rates for both household and health facility interviews (85.9% and 77.8% respectively).

A total of 122 health facilities and 122 patients participated in both phone interviews and in-person validation interviews between December 2016 and December 2017. Table 3 summarizes the key findings on the validity of data from phone interviews. At the health facility level, there was a strong and statistically significant level of agreement between medicine availability (kappa = 0.90; CI 0.89–0.92); and a strong intra-class correlation between prices of medicines (ICC = 0.96; CI 0.95–1.0), comparing data from phone and in-person interviews. The mean difference between price data collected via in-person and phone interview was 0.05 times the standard deviation (of in-person data). Supplementary Appendix S5 shows the Bland-Altman plot of price data collected through phone and in-person interviews at health facilities. The level of agreement on whether the recommended pack size of a medicine was available or not was also strong.

**Table 3:** Key findings on validity of data from phone interviews

	Household		Health facility	
	Phone	In-person	Phone	In-person
Number of interviews	130	130	123	123
Response rates (%)	94.5		88.2	
Mean interview duration in minutes	12.8	8.3	30.9	14.9
Agreement between availability reported over the phone and in-person	kappa = 0.49 (CI: 0.39–0.60) *N* = 173		kappa = 0.90 (CI 0.88–0.92) *N* = 3748	
Agreement between price data reported over the phone and in-person^a^	ICC = 0.47 (CI 0.34–0.57) *N* = 177		ICC = 0.96 (CI 0.95–0.96) *N* = 731	
Agreement between quantity of medicines purchased reported over the phone and in-person^a^	ICC = 0.4 (CI 0.27–0.52) *N* = 182		N/A	
Agreement between strength of medicines reported over the phone or in-person^a^	ICC = 0.99 (CI 0.99–1.00) *N* = 118		N/A	
Agreement between place of purchase reported over the phone and in-person	Kappa = 0.53 CI (0.43–0.62) *N* = 185		N/A	
Agreement between the availability of recommended pack sizes of medicines in facility (Yes/No)	N/A		Kappa = 0.89 (CI 0.85–0.93) *N* = 712	

N/A, not applicable; ICC, intra-class correlation coefficient.

a The means of the differences between phone data and in-person data for price at the facility level, price at the household level, quantity purchased, and strength of medicine were 0.05, 0.50, 0.64 and 0.03 times the standard deviations (of in-person data).

At the household level, there was a moderate level of agreement between medicine availability (kappa = 0.49, CI: 0.39—0.60); and a moderate intra-class correlation between prices of medicines (ICC = 0.47, CI 0.34—0.57), comparing data from phone and in-person interviews. The intra-class correlation for the strength of medicines purchased was strong (ICC = 0.99), while the correlation for the quantity of medicines purchased was moderate (ICC = 0.4). The means of the differences between in-person data and phone data for price, strength, and quantity purchased were 0.50, 0.03 and 0.64 times the standard deviations (of in-person data) respectively. The Bland-Altman plot of phone and in-person data on price, quantity and the strength of medicine purchased at the household level are shown in Supplementary Appendix S5. From the Bland-Altman statistics, the mean difference for phone and in-person data on price was relatively large (255.36 KES) with wide 95% limits of agreement (−1516.04 to 2026.76) (Supplementary Appendix S5e). The level of agreement between the place of purchase reported over the phone and in-person was moderate (Kappa = 0.53).

### Impact of phone surveillance on behaviour

There was no evidence of the impact of phone surveillance on the probability of having at least one medicine at home (*P* = 0.75; Table 4).

**Table 4:** —Impact of phone surveillance on availability of medicines in households.

	Not surveilled (1 year)	Surveilled (1 year)	OR (95% CI) adjusted	*P*-value
Probability of having at least 1 medicine at home (at midline)	162 (81%)	296 (79.8%)	0.93 (0.57–1.49)	0.75
*N*	200	371		

### Costing of survey modalities

Table 5 displays the costs associated with each mode and type of data collection. The cost per phone interview at health facilities and households were $19.73 and $16.86 respectively, which is much lower than the cost per in-person interview at the baseline of $186.20. Each in-person validation interview cost $38.84. The cost of listing eligible health facilities and households (which preceded baseline data collection) was $48 175.31. The breakdown of these costs is presented in Appendix 6.

**Table 5:** Costs of phone interviews with in-person interviews

	Recurrent costs		Start-up costs
Costs	Phone calls (facility)	Phone calls (Household)	Baseline (in-person)
Number of interviews conducted	138 (per month)	430 (per 3-month cycle)	1020 (All interviews at baseline)
Cost per interview (total cost/no. of interviews)	$19.73	$16.86	$186.20
Total number of interviews per year	1656	1720	
Total cost of interviews per year	$32 666.40	$28 997.76	$189 927.27 (one-off cost)

### Qualitative results

A total of 24 IDIs were conducted. This included IDIs with six data collectors, and nine each of health facility and household study participants.

The majority of IDI participants believed that the data generated through phone interviews were accurate compared to data from in-person interviews. The facilitators and challenges that are common to phone interviews and in-person interviews and the facilitators and challenges that are unique to each of these modes of data collection are summarized in Table 6.

**Table 6:** Perceived facilitators and challenges to in-person and phone data collection

Method	Facilitators	Barriers
In-person and phone interviews	Village elders helped data collectors locate households of patients, and also serve as interpreters When DCs and respondents speak the same language Having multiple contact information of respondents Familiarity of respondents with medicine terminologies Scheduling appointments with respondents in advance	Language barrier Busy schedule of respondents (especially health facilities) Respondents having limited or no understanding of the purpose of the study Limited trust between respondents and DCs. Some variables were particularly challenging to collect data on: price, strength of medicine, place of purchase, pack size of medicine
In-person interviews only	Facial contact between data collectors and respondents Ability of FOs to visually confirm the medicine and the data collected	Poor road networks Bad weather Time constraints Relocation of study participants Households not close to each other
Phone interviews only	Familiarity with data collection over the phone Relatively low cost Less time consuming	Poor phone network Inaccurate phone numbers or respondents changing their numbers Hard to tell if respondents are giving accurate data

#### Facilitators of in-person and phone data collection

Several of the facilitators reported for data collection in-person and via phone interviews were similar. Village elders played multiple roles in both in-person and phone interviews, including helping to locate households of patients, facilitating good rapport between data collectors and patients, and serving as interpreters.

*Most of the time I use the village elder to go to this particular respondent's home to explain to them why I want to talk to them, because sometimes for these people that do not understand Swahili very well and they are so used to talking in another tongue. The moment I greet them in Swahili they will tell me wrong number…so in this case I will tell the guide [village elder] to go to this respondent's home and talk to the respondent and explain why I was calling.* (Participant FO01—data collector)

Some of the facilitators discussed were unique to in-person interviews, including the ability of data collectors to visually confirm the medicines at the household or facility level. The flexibility of scheduling interviews, low cost and the relatively less time involved were among the facilitators described for phone interviews.

#### Challenges associated with data collection via in-person and phone interviews

As was in the case of facilitators, several of these challenges were common to both modes of data collection. Language barrier and limited familiarity with medical terminology were the biggest challenges at the household level where respondents were less educated. Busy schedule of respondents (particularly at the facility level), and the fear of respondents that the data collected may be used for other purposes unknown to them were some of the other common challenges.

*So there is that some somehow some fear, some lack of trust because now I am giving the prices of how much we sell, how much we buy, the name of the drugs we have,…you might think thieves can know I stock these palliative drugs … so somehow there is that mistrust as you give the information. (*IDI HFM04, health facility on phone interviews*)*

Irrespective of the mode of data collection, variables such as price, the strength of medicine, place of purchase, pack size of medicine and differentiating between generic and originator brands were reported as challenging to collect data on. The reasons for the difficulty include poor labelling on medicines dispensed to patients, respondents from lower-level facilities not having adequate knowledge on medications, recall difficulties, and low literacy level of some respondents. More specifically, it was difficult to collect price information in cases where patients did not purchase the medicines themselves or the bought the NCD medicines of interest together with other medicines.

A number of challenges were uniquely associated with in-person data collection (poor road networks, bad weather, time constraints etc.) and telephone data collection (poor or inconsistent telephone network in some areas, not having the correct phone number etc.). To overcome the challenge of the poor phone network, data collectors and their respondents used additional means of communication. For example, facilities were sent a form (by email, SMS or WhatsApp) and respondents filled in the requested information and then sent it back when they regained connectivity.

## Discussion

This study has generated useful findings on the feasibility of using phone surveys to monitor and evaluate public health policies and programs at household and health facility levels. These findings and their implications are discussed below.

### Validity of data from phone interviews

To the best of our knowledge, this is the first study to compare the validity of medicine price and availability data collected from phone interviews with in-person interviews. For both primary outcomes, price and availability of medicines, data collected over the phone showed moderate to strong validity with data collected in-person. Though not specific to data collection on medicines, several studies have demonstrated the validity of data collected via phone interviews (Roccaforte *et al.*, 1992; Barrington *et al.*, 2010; Janssen *et al.*, 2010; Hajebi *et al.*, 2012; Githinji *et al.*, 2013; Szolnoki and Hoffmann, 2013; Silva *et al.*, 2014). Our findings suggest that telephone data collection may be more feasible at the facility level where there was a strong validity of data collected on all variables evaluated: availability, price and availability of recommended pack size.

Except for phone data on strength of medicine, which showed a strong validity, data on variables such as availability, price and quantity of medicine purchased showed only moderate validity at the household level. For the price, this moderate validity is corroborated by the wide limits of agreement observed in the Bland–Altman plot (Supplementary Appendix S5b and e). Based on the findings from our qualitative interviews, several factors may be responsible for the relatively low validity of telephone data at the household level. These include poor labelling of medicines dispensed to patients, low literacy level of some patients and recall difficulties. More specifically regarding price data, some patients did not purchase the medicines themselves and therefore could not remember the price, while others bought their NCD medicines together with other medicines and could not tell how much each medicine cost. These challenges are likely to impact data collection more at the household level. To improve validity of data collected at the household level, additional functionalities of mobile phones could be explored including video calls where data collectors can see patient medicines virtually and sharing pictures of the medicine labels and packages as part of telephone data collection.

The high response rates obtained (88.2% for health facilities and 94.5% for households) may be due to the incentives provided [household and facility respondents received 50 KES ($0.5) and 100 KES ($1) worth of airtime, respectively] during each data collection (Croke *et al.*, 2012; Ballivian *et al.*, 2015). We also started telephone surveillance about two months after baseline in-person data collection when the study was introduced to respondents. Thus, surveillance started when respondents were less likely to have forgotten about the study or lost their motivation to participate (Dillon, 2010). Though not regarding the collection of data on medicines, other phone surveys have reported response rates of close to 90% and above (Barrington *et al.*, 2010; Githinji *et al.*, 2013; Etang-Ndip *et al.*, 2015). More specifically, the response rate of 88.2% at the facility level is consistent the response rate of 87.5% achieved in collecting data on medicines availability in pharmacies and hospitals in Madagascar (Jost *et al.*, 2016). The lower response rates observed at the facility level compared to households may be due to the busy schedules of facility respondents as discussed in our qualitative interviews. Additionally, data collection took longer, especially for phone interviews at facilities compared to households (30.9 vs 12.8 min) which may have affected response rates at facilities. At the facility level, data were collected on at least 31 medicines (and for each medicine the originator brand, lowest priced generic and *Novartis Access* brand where available) compared to households where data were collected on an average of two to three medicines per patient.

There is the possibility that routinely calling patients to collect data on their medicines may affect their medicine seeking behaviour. Phone calls may remind patients to purchase their medicines. However, in a randomized sub-study, we did not find any impact of the phone surveillance on availability of medicines at the household level (*P* = 0.751; Table 4). This further emphasizes the validity of the phone interviews as a tool for research and surveillance data collection.

### Cost of phone and in-person interviews

The cost estimates found in this study are similar to what has been documented in the literature in LMICs. Ballivan et al reported the cost per CATI of $25 for household surveys, while $22.2 per phone interview was reported in another study (Ballivian *et al.*, 2015; Mahfoud *et al.*, 2015). These costs are a little higher than the $19.73 and $16.86 (for health facilities and household respectively) found in our study (Ballivian *et al.*, 2015). Some studies in Tanzania however, reported lower costs ($4.10 to $7.30 per phone interviews) (Dillon, 2010; Croke *et al.*, 2012).

Most importantly, this study has also shown that phone interviews cost much less than in-person interviews both in households and health facilities. The cost per in-person interview at baseline was at least 10 times more than the costs per phone interview at households and health facilities. Mahfoud *et al.* (2015) showed that phone data collection saved about $14 per interview, a much smaller difference compared to our study. Despite the lower cost of telephone interviews, there may be the need to conduct in-person interviews at baseline partly in order to collect phone numbers of study respondents and also inform them that they would be called. This means the cost associated with initial baseline in-person data collection may not be avoided unless there is an existing unbiased source of telephone numbers such as patient registries (Croke *et al.*, 2012; Ballivian *et al.*, 2015; Himelein and Kastelic, 2015). Alternatively, phone numbers could be collected during the listing of households and health facilities. In this study, listing costs were much lower than baseline in-person data collection costs (48 175.31 vs 189 927, respectively). Telephone interviews alone or in combination with in-person baseline data collection or household listing presents a cost-effective opportunity for researchers, program implementers and policy evaluators to collect good quality baseline data and to identify respondents.

The incentives given to respondents in this study represent a very small fraction of the total cost for each interview. This shows that incentives can be provided to potentially increase response rates without substantially increasing the overall budget for telephone data collection.

### Perceptions of data collectors and respondents on telephone and in-person interviews

Responses from IDIs uncover the perceptions of data collectors and participants in the *Novartis Access* evaluation on providing or collecting data via in-person and phone interviews.

Several of the facilitators reported by study participants were similar for both in-person and phone interviews. The most prominent of these was the role of village elders in helping locate study participants, building trust between the participants and data collectors and also serving as interpreters. This shows that irrespective of the mode of data collection, additional help may be needed by data collectors, either remotely or in the field. The main facilitator unique to in-person interviews is that data collectors were able to confirm the data collected, observe body language and facial expressions of the respondents and in some cases assist illiterate participants in reporting information. Observing the nonverbal communication aspects are important during interviews (Dillon, 2010). The reported facilitators that are unique to phone data collection include its low cost and less time-consuming nature. Though not part of the original design of the study, data collectors in some cases used communication via SMS and WhatsApp to navigate the challenges of poor network.

The key challenges reported for both in-person and phone data collection were also similar -language barrier, and the busy schedule of respondents. While language barriers were reported as a challenge for both modes of data collection, this barrier could be potentially addressed for phone data collection—in which data collectors with different language skills call respondents from a call centre. Another way of addressing language barriers, which was the case in this study, is the use of translators or a village elder to serve as a translator. This approach may take more time or invade the privacy of primary respondents. It could also lead to social desirability bias, translation errors and increased cost (Dillon, 2010). Some of the challenges associated with in-person data collection, such as poor road networks, bad weather and relocation of study participants can substantially hinder data collection. In these cases, it may be worth considering data collection via phone interviews. Two important challenges associated with phone data collection mentioned were poor telephone network coverage and limited power supply. However, these challenges seem not to have affected the high response rates obtained. Nonetheless, inconsistent network coverage is a potential source of bias. Network coverage is likely to be correlated with wealth, distance from major towns and access to other utilities like water supply (Dillon, 2010; Himelein and Kastelic, 2015). Therefore network coverage should be considered in the design and location of phone surveys (Demombynes *et al.*, 2013; Etang-Ndip *et al.*, 2015). Charging stations could also be provided to ensure that respondents charged their phones to be able to provide data (Dillon, 2010).

The fact that household respondents had more problems with language barrier and more difficulty understanding the study and medical terminology, may mean that in-person data collection may be less feasible at the household level. As mentioned earlier, this may be one of the reasons for the sub-optimal validity of phone data collected at the household level. Depending on the technology available, and how familiar respondents are with phone use, other functionalities of phone surveys, such as sharing of pictures or videos can be explored to address these barriers.

### Study limitations

All the participants in this study had access to a phone and the telecommunication network and the supply of electricity in the study counties were generally stable. These may in part have contributed to the high response rates for telephone interviews. Thus, the high response rates obtained in this study may not be generalizable to other parts of Kenya and countries with low phone ownership or very poor network or limited supply of electricity. Additionally, the in-person baseline data collection that preceded the telephone surveillance might have increased the familiarity and trust between data collectors and respondents. This might also have also increased the willingness of respondents to participate in the surveillance and the provision of accurate data. Other strategies may be needed to increase response rates in cases where in-person baseline data collection did not precede data collection via phone interviews. Furthermore, this baseline data collection provided the telephone numbers to call. In circumstances where it is not possible to collect the phone numbers of respondents during baseline data collection, and there is no unbiased source of phone numbers available, phone numbers and consent to call may need to be collected during medical consultations or at the point of dispensing. The generalizability of cost estimates from this study may also be limited by the potential variations in the costs associated with telephone and in-person data collection across countries.

## Conclusion

Many policy or programmatic interventions are undertaken by public, private and non-governmental organizations but these are frequently not evaluated (Rockers *et al.*, 2017). This shortcoming is often justified by the cost and complications of undertaking a rigorous methodologically sound evaluation. This study demonstrated that telephone interviews can yield high response rates and valid data at a low cost. While the validity of data from telephone interviews compared to in-person interviews was strong at health facilities, the validity observed at the household level was only moderate. More needs to be done to increase the validity of household data collected via phone interviews. Most of the facilitators and challenges for phone and in-person data collection are similar. Utilizing telephone interviews in countries with high cell phone ownership and distribution provides an opportunity for relatively low cost validated data collection for randomized control trials or interrupted time series evaluations.

## Supplementary data

Supplementary data are available at *Health Policy and Planning* online.

## Funding

This work was supported by Sandoz International GmbH. The data collection, analysis, writing and publications of the results are not subject to control by the funding organization (see also agreement http://sites.bu.edu/novartisaccessevaluation/agreements/).

*Conflict of interest statement:* None declared.

*Ethical approval:* This research study was reviewed and approved by the Institutional Review Boards of the Boston University Medical Campus and Strathmore University in Kenya.

## Supplementary Material

czab029_Supp
