# Supplementary material for: Phone-based monitoring to evaluate health policy and program implementation in Kenya
Source: Health Policy Plan. 2021 Mar 16;36(4):444–53. doi: 10.1093/heapol/czab029 (PMC8128015; doi:10.1093/heapol/czab029)
Supplement: czab029_Supp [file czab029_supp.zip › Appendices.docx]

**Appendix 1: List of medicines rotated during each month of facility data collection**

| **Group 1** | **Group 3** |
| --- | --- |
| Amitriptyline 25mg Tablet/Capsule | Captopril 25mg Tablet/Capsule |
| Amoxicillin 250mg Tablet /Capsule | Ceftriaxone 1 g/vial Injection |
| Amoxicillin 500mg Tablet/Capsule | Ceftriaxone 250mg/vial Injection |
| Glibenclamide 5mg Tablet/Capsule | Co-trimoxazole 8+40mg/ml Suspension |
| Omeprazole 20mg 20 mg Tablet/capsule | Paracetamol 24 mg/ml Suspension |
| **Group 2** |  |
| Atenolol 50mg Tablet/Capsule |  |
| Ciprofloxacin 500mg Tablet/Capsule |  |
| Ciprofloxacin 250mg Tablet/Capsule |  |
| Diazepam 5mg Tablet/Capsule |  |
| Diclofenac 50mg Tablet/Capsule |  |
| Diclofenac 100mg Tablet/Capsule |  |

**Appendix 2: Duration of facility interviews (in minutes) by month of data collection**

| Month | Telephone interviews | | | | | Validation visits | | | | |
| --- | --- | --- | --- | --- | --- | --- | --- | --- | --- | --- |
|  | N | Mean | Median | Minimum | Maximum | N | Mean | Median | Minimum | Maximum |
| 1 | 6 | 22.5 | 22.3 | 9.6 | 37.5 | 6 | 16.7 | 11.3 | 9.2 | 45.4 |
| 2 | 10 | 28.4 | 25.9 | 4.3 | 56.1 | 11 | 18.7 | 15 | 6.7 | 56.1 |
| 3 | 12 | 30 | 21.5 | 7.8 | 69.6 | 10 | 18.4 | 9.9 | 4.9 | 87.7 |
| 4 | 11 | 27.2 | 23.4 | 12.8 | 53.9 | 12 | 10.9 | 9.1 | 4 | 25 |
| 5 | 24 | 32.2 | 26.2 | 9.4 | 90.4 | 25 | 14.5 | 9.3 | 4.9 | 107.5 |
| 7 | 8 | 30.5 | 25.7 | 8.1 | 58.8 | 8 | 13.5 | 11.2 | 4 | 34.6 |
| 8 | 8 | 39.6 | 27.4 | 15.1 | 87 | 7 | 9 | 6.8 | 3 | 19.1 |
| 9 | 7 | 38.4 | 41 | 19.3 | 61.2 | 8 | 17.8 | 15.6 | 8.1 | 38.7 |
| 10 | 8 | 25.5 | 19.7 | 8.8 | 53.7 | 8 | 11.7 | 8.9 | 5.2 | 26.5 |
| 11 | 7 | 30.5 | 26.7 | 9.2 | 53 | 8 | 15.2 | 10.8 | 3.3 | 43.1 |
| 12 | 8 | 37.7 | 27.6 | 14.1 | 102.5 | 8 | 19 | 12.3 | 5 | 73.9 |
| 13 | 7 | 27.2 | 22.7 | 7.6 | 88.3 | 6 | 13.4 | 9.1 | 4.2 | 34.1 |
| **Overall** | **116** | **30.9** | **25.1** | **4.3** | **102.5** | **117** | **14.9** | **10.5** | **3.0** | **107.5** |

**Appendix 3: Duration of household interviews (in minutes) by month of data collection**

| Month | Phone interviews | | | | | Validation visits | | | | |
| --- | --- | --- | --- | --- | --- | --- | --- | --- | --- | --- |
|  | N | Mean | Median | Minimum | Maximum | N | Mean | Median | Minimum | Maximum |
| 1 | 5 | 7.7 | 6.7 | 4.2 | 13.9 | 5 | 10.3 | 7.9 | 3.5 | 25.4 |
| 2 | 12 | 10.2 | 7 | 3.2 | 32.8 | 12 | 8.8 | 6.9 | 2.5 | 19.8 |
| 3 | 14 | 11.4 | 8.7 | 5.3 | 30.9 | 14 | 10.2 | 7.4 | 1.6 | 28 |
| 4 | 14 | 8.7 | 7.6 | 1.7 | 21.7 | 13 | 9.2 | 9.6 | 4.1 | 16.8 |
| 5 | 12 | 10.8 | 7.7 | 2.4 | 44.9 | 12 | 8.2 | 7 | 1.6 | 18.5 |
| 6 | 15 | 8.5 | 6.7 | 2 | 15.6 | 15 | 7.9 | 5.6 | 1.7 | 30.1 |
| 7 | 9 | 14 | 8.3 | 2.4 | 36.8 | 9 | 5.5 | 5.4 | 1.4 | 16.1 |
| 8 | 9 | 15.9 | 8.4 | 3.4 | 42.3 | 8 | 7.8 | 9.4 | 1.7 | 12.9 |
| 9 | 8 | 16.1 | 13 | 8.4 | 30.5 | 8 | 10.3 | 7 | 3.9 | 30.2 |
| 10 | 8 | 14.7 | 14.6 | 2.3 | 28.2 | 8 | 6.8 | 5.6 | 3.6 | 15.8 |
| 11 | 10 | 22.8 | 22.8 | 4.3 | 54.2 | 10 | 7.9 | 8.2 | 2.9 | 15.6 |
| 12 | 8 | 20.2 | 20 | 3.8 | 33.8 | 8 | 5.5 | 2.4 | 0.7 | 17.5 |
| 13 | 6 | 9.4 | 5 | 3.4 | 21.5 | 6 | 7.8 | 6.5 | 2.3 | 16.7 |
| **Overall** | **130** | **12.8** | **9.3** | **1.7** | **54.2** | **128** | **8.3** | **6.7** | **0.7** | **30.2** |

**Appendix 4: Response rates (%) by county**

|  | **Embu** | **Kwale** | **Kakamega** | **Makueni** | **Narok** | **Nyeri** | **Samburu** | **West Pokot** |
| --- | --- | --- | --- | --- | --- | --- | --- | --- |
| Household | 98.9 | 94.5 | 93.7 | 98.5 | 87.2 | 96.0 | 85.9 | 89.8 |
| Health facility | 91.1 | 92.7 | 85.8 | 91.2 | 84.4 | 86.1 | 77.8 | 94.6 |

| **Appendix 5. Bland-Altman plots of the differences between telephone and in-person data (vertical axis) and the means of telephone and in-person data (horizontal axis)** | |
| --- | --- |
| 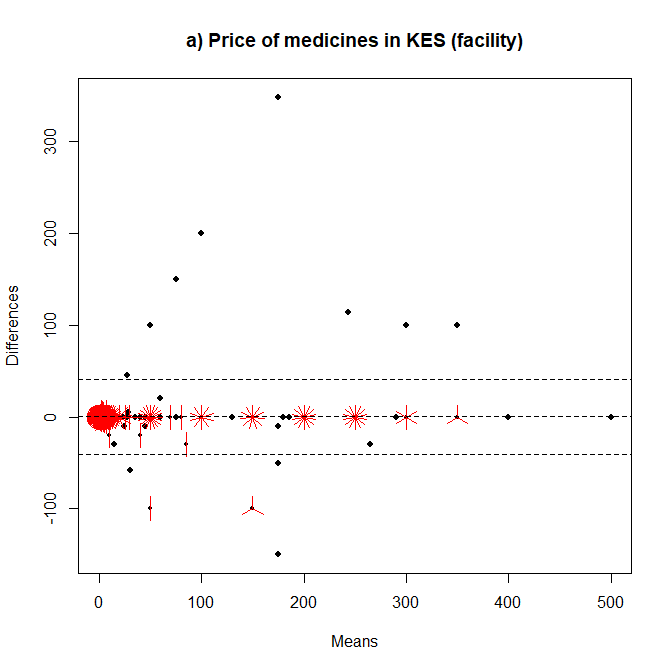 | 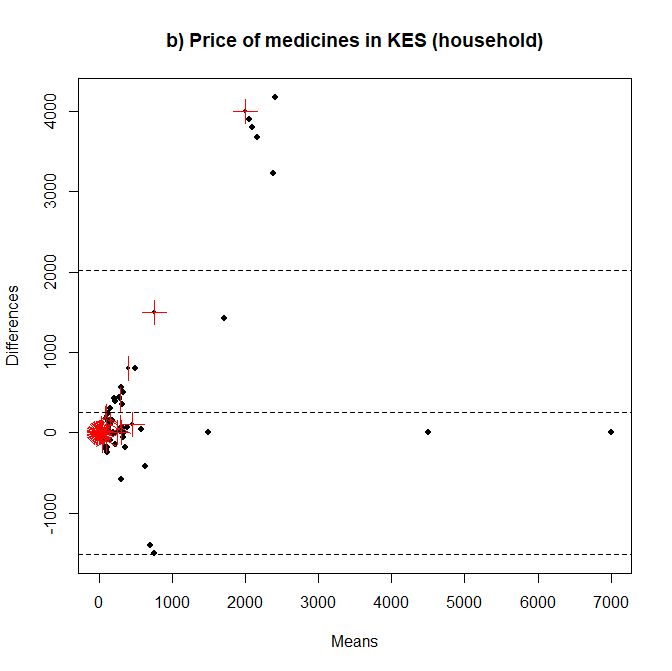 |
| 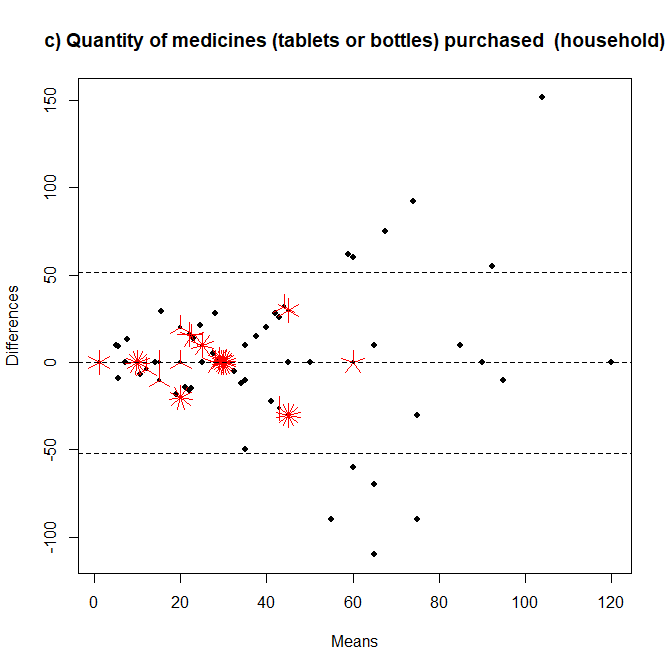 | 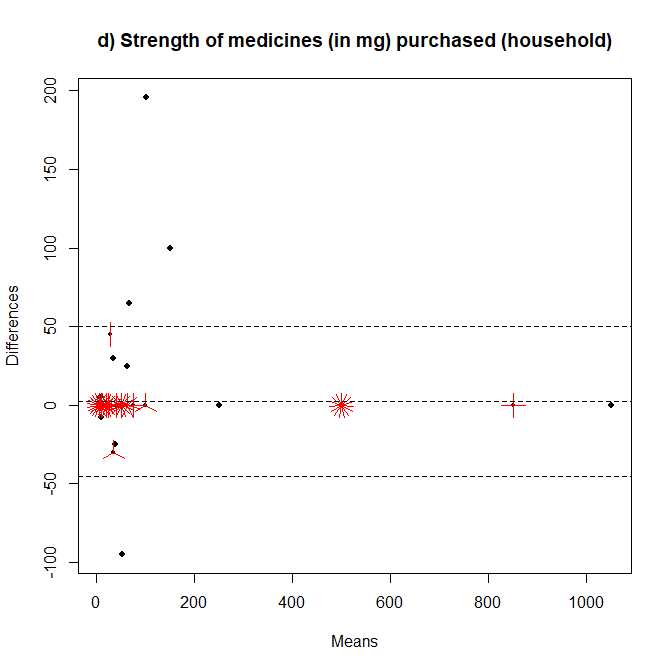 |

*Lines represent the mean difference between telephone and in-person data, and the lower and upper limits of the 95% Limits of Agreement; The sunflowers show ties in the data; KES = Kenyan Shillings.*

**e) Summary of Bland-Altman statistics**

| ***Variable*** | ***Mean difference between telephone and in-person data*** | ***95% Limit of agreement between telephone and in-person data*** |
| --- | --- | --- |
| Price of medicines in KES (health facility) | 0.27 | -40.41 – 40.94 |
| Price of medicines in KES  (household) | 255.36 | -1516.04 – 2026.76 |
| Quantity of medicines (# of tablets/bottles) purchased (household) | -0.18 | -51.98 – 51.62 |
| Strength of medicine in mg (household) | 2.49 | -45.43 – 50.40 |

**Appendix 6: Comparing costs of phone interviews with in-person interviews**

| ***Costs*** | ***Phone calls (facility)*** | ***Phone calls (household)*** | ***In-person validation visits: Household & facility*** | ***Baseline (in-person)*** | ***Listing/***  ***Piloting*** |
| --- | --- | --- | --- | --- | --- |
| Assessor staff salaries | $580 | $1,740 | $580 | $23,065 | $3,091 |
| Management staff salaries | $850 | $2,550 |  | $59,250 | $11,307 |
| Respondent gifts | $276 | $215 | $30 | $2,169 | - |
| Transportation | - | - | $200 | $10,471 | $655 |
| Airtime | $57 | $156 | $12 | $1,825 | $40 |
| Other costs |  |  |  |  |  |
| Occupancy (desk space) | $160 | $480 |  | $350 | $2,650 |
| Equipment (computers, phones for managers, tablets, bags, notebooks, rain gear,) | $63 | $55.24 | $4.62 | $18,300 |  |
| Software (computer-assisted software): | $4 | $11 |  | $238 |  |
| Field guides |  | *$90* |  | $3,062 |  |
| Accommodation (in-person data collection) |  |  | $160 | $19,441 |  |
| Training location cost |  |  |  |  | $688 |
| Printing costs |  |  | $35.00 | $614.00 | $341 |
| Cost of all the supplies and equipment before project begun: |  |  |  |  | $16,431 |
| *Total direct costs* | *$1,989* | *$5,297* | *$1,022* | *$138,785* | *$35,203* |
| *Administrative costs - assume 19% of all other direct costs* | *$377.95* | *$1,006.50* | *$194.11* | *$26,369.15* | *$6,688.57* |
| *Total direct + administrative costs* | *$2,367.15* | *$6,303.86* | *$1,215.73* | *$165,154.15* | *$41,891.57* |
| *Over-head – 15% of direct including administrative costs* | *$355.07* | *$945.58* | *$182.36* | *$24,773.12* | *$6,283.74* |
| **Grand total** | **$2,772.22** | **$7,249.44** | **$1,398.09** | **$189,927.27** | **$48,175.31** |
| **Interviews** |  |  |  |  |  |
| Number of interviews conducted (per month for facilities and per 3months for households) | 138 | 430 | 36 | 1020 |  |
| **Cost per interview (Total cost/# interviews)** | **$19.73** | **$16.86** | **$38.84** | **$186.20** |  |
| Total number of interviews per year | 1656 | 1720 | 432 |  |  |
| **Total cost of interviews per year** | **$32,666.4** | **$28,997.76** | **$16,777.08** |  |  |
